# Insulin-Like Growth Factor-1 (IGF-1) and Its Monitoring in Medical Diagnostic and in Sports

**DOI:** 10.3390/biom11020217

**Published:** 2021-02-04

**Authors:** Julian Bailes, Mikhail Soloviev

**Affiliations:** Department of Biological Sciences, Royal Holloway University of London, London TW20 0EX, UK

**Keywords:** insulin-like growth factor, IGF, IGFBP, IGF-1R, IGF-2R, IGFBP-rP, IGFBPL1, ALS, insulin, doping

## Abstract

Insulin-like growth factor-1 (IGF-1) is the principal mediator of growth hormone (GH), plays a crucial role in promoting cell growth and differentiation in childhood and continues to have an anabolic effect in adults. IGF-1 is part of a wide network of growth factors, receptors and binding proteins involved in mediating cellular proliferation, differentiation and apoptosis. Bioavailability of IGF-1 is affected by insulin-like growth factor binding proteins (IGFBPs) which bind IGF-1 in circulation with an affinity equal to or greater than that of the IGF-1 receptor (IGF-1R). The six IGFBPs serve as carrier proteins and bind approximately 98% of all circulating IGF-1. Other proteins known to bind IGF-1 include ten IGFBP-related proteins (IGFBP-rPs), albeit with lower affinities than the IGFBPs. IGF-1 expression levels vary in a number of clinical conditions suggesting it has the potential to provide crucial information as to the state of an individual’s health. IGF-1 is also a popular doping agent in sport and has featured in many high-profile doping cases in recent years. However, the existence of IGFBPs significantly reduces the levels of immunoreactive IGF-1 in samples, requiring multiple pre-treatment steps that reduce reproducibility and complicates interpretation of IGF-1 assay results. Here we provide an overview of the IGF network of growth factors, their receptors and the entirety of the extended family of IGFBPs, IGFBP-rPs, E peptides as well as recombinant IGF-1 and their derivatives. We also discuss issues related to the detection and quantification of bioavailable IGF-1.

## 1. Introduction

### 1.1. IGF-1: Structure, Function and Mode of Action

**Figure 1:**
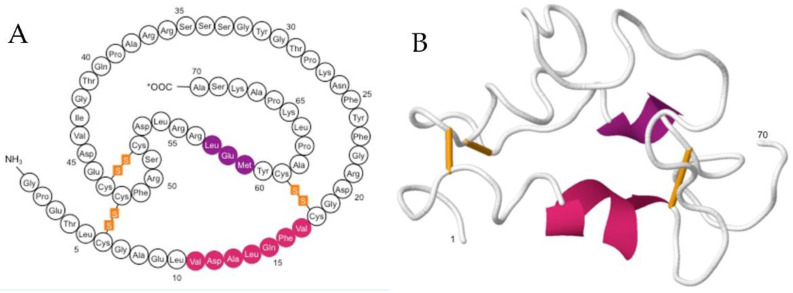
Structure of human Insulin-like growth factor-1 (IGF-1). (**A**) Primary structure showing disulphide bridge between amino acids 6 and 48, 18 and 61 and 47 and 52.; (**B**) 3D structure of IGF-1 showing folding of the polypeptide chain and secondary structural elements as ribbons (PDB ID: 1BQT).

Insulin-like growth factor-1 (IGF-1) is a 70 amino-acid single chain peptide with a molecular weight of 7.6 kDa. IGF-1 contains three disulphide bridges, between amino acids 6 and 48, 18 and 61 and 47 and 52, which create the tertiary structure critical for optimum binding to the IGF-1R (Figure 1). As its name suggests, IGF-1 is structurally similar to insulin and is capable of binding to the insulin receptor (I-R), albeit with a lower affinity than that towards IGF-1R. IGF-1 is the principal mediator of growth hormone (GH), it plays a crucial role in promoting cell growth and differentiation in childhood and continues to have an anabolic effect in adults. When GH is produced by the anterior pituitary gland its release into the bloodstream stimulates the production and release of IGF-1, which subsequently binds to the IGF-1R displayed on the surface of nearly every cell in the body. IGF-1R is comprised of two alpha and two beta subunits linked by disulphide bonds. The transmembrane beta subunits each have intracellular tyrosine kinase domains that are activated upon IGF-1 binding to the extracellular alpha subunits. Activation of these kinase domains results in the activation of multiple signaling pathways including the PI3K/Akt pathway and the Raf/MEK/ERK cascade that ultimately prevent apoptosis and promote cellular growth and survival [1]. IGF-1 and its receptor IGF-1R are involved in these critical pathways and therefore play a pivotal role in healthy tissue homeostasis but also a far less desirable role in diseases such as cancer where they can be responsible for the survival and proliferation of malignant cells. IGF-1 acts mostly as an endocrine hormone secreted primarily by the liver [2,3] and transported to the target tissues. It is also produced by tissues outside the liver where it acts locally in a paracrine fashion and is believed to play an important autocrine role in cancer [4]. 

The full length IGF-1 precursor protein (P05019) has a signal peptide (pos. 1–21), a propeptide (pos. 22–48), an IGF-1 sequence (pos. 49–118) and an E peptide sequence (pos. 119–195). Alternative splicing of IGF-1 mRNA yields three pro-hormones or so called “E” peptides [5]. There is increasing evidence that these E-peptides (Ea, Eb, Ec) themselves have functional roles that are independent of IGF-1 receptor activation, including effects on proliferation and cell migration. Pfeffer et al. [6] found that Ea and Eb peptides enhance the uptake of IGF-1 into cells or aid in the release of IGF-1 from IGFBPs and its binding to IGF receptors. Hede et al. [7] reported a tethering function of E peptides that could aid in maintaining concentrations of IGF-1 at their site of synthesis to enable more localized effects. The IGF-1 Ec peptide, also known as mechano-growth factor (MGF), is up-regulated in exercised and damaged muscles, plays a neuroprotective role against ischemia and contributes to the actions of IGF-1 to improve cardiac function and activate resident stem cell populations [8,9,10,11,12,13,14,15]. Exogenous administration of synthetic Ec peptides leads to phosphorylation of extracellular signal regulated kinases (ERK1 and ERK2), serine/threonine-specific protein kinase (Akt), IGF-1 and insulin receptor-independent cell growth [16] and produces mitogenic effect on human cell lines [17,18].

### 1.2. Growth Factors and IGFBPs

**Figure 2:**
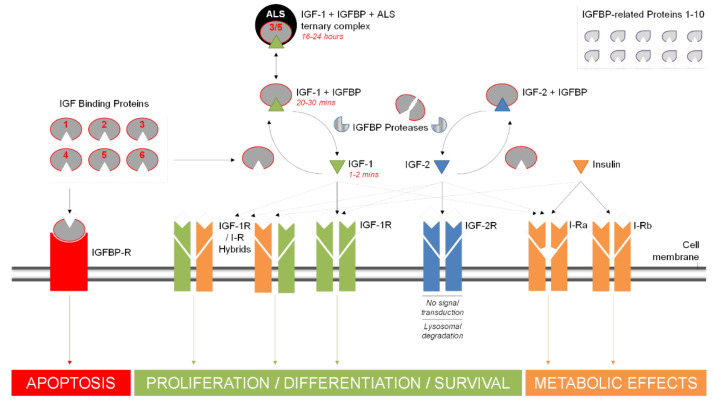
Insulin-like growth factor-1 (IGF-1) is part of a wider network of growth factors, receptors and binding proteins involved in mediating cellular proliferation, differentiation and survival. In addition to IGF-1 two other polypeptides, Insulin-like growth factor-2 (IGF-2) and insulin feature prominently, as well as multiple membrane-bound receptors including the type-1 and type-2 IGF receptors (IGF-1R and IGF-2R), two insulin receptor isoforms (I-Ra and I-Rb), a number of hybrid receptors between the insulin and IGF-1 receptors (IGF-1R/I-R) and the two I-R isoforms themselves (not shown). All three peptides bind to their respective receptors with the highest affinity (denoted by solid black arrows) but also share some crossover affinity with each other’s receptors (dashed arrows). The bioavailability of IGF-1 and IGF-2 is regulated by a group of six insulin-like growth factor binding proteins (IGFBPs) that chaperone the peptides in circulation. A majority of IGF-1 is bound to IGFBP3 or 5 and the acid-labile subunit (ALS), forming a ternary structure known as the IGF-1-IGFBP-ALS complex. Half-life times for bound and unbound IGF-1 are shown in red italics. Cell surface interactions or IGFBP proteolysis frees IGF-1 to bind its receptor. IGF-2 further regulates IGF-1 signalling by binding to the IGF-1R. Whilst the IGF-2R does not participate in signalling, it does lead to lysosomal degradation of IGF-2, thus reducing competition for the IGF-1R when required. IGFBPs also show an IGF-independent role, binding to a potential IGFBP receptor (IGFBP-R) and promoting apoptosis via signalling pathways and/or intracellular activity after internalisation by the cell. IGFBP-related proteins (IGFBP-rPs, shown top right) are another class of molecules involved in the IGF network with affinity for IGFs, albeit at lower affinities than the IGFBPs.

**Figure 3:**
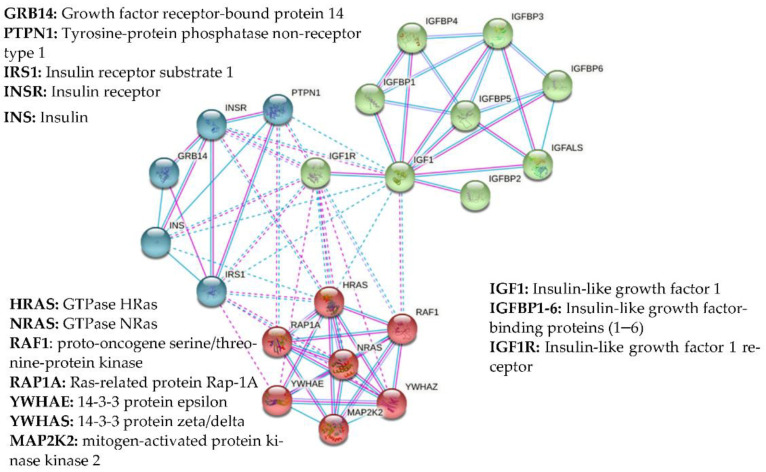
Network of experimentally confirmed (purple lines) and documented (blue lines) protein-protein interaction (PPI) of insulin-like growth factor-1 (IGF-1) generated with string-db.org. Direct physical protein-protein interactions with IGF-1 indicated here are based on the reported experimental evidence from chemical crosslinking experiments, immunoprecipitation and size exclusion chromatography studies [31], ^121^I-labelled ligand binding [32], real-time label-free binding [33] and x-ray crystallography studies [34,35]. Known functional interactions are based on the information from Reactome Pathways (www.reactome.org), Gene Ontology Consortium (www.geneontology.org) and manually curated metabolic and signalling pathways (www.genome.jp/kegg/pathway). The network was clustered to the specified number of clusters (*n* = 3) using kmeans clustering. Proteins clustered together are shown with particular colours, intra-cluster PPIs with solid lines, inter-cluster PPIs with dashed lines.

Insulin-like growth factor-2 (IGF-2) is a protein closely related to IGF-1 and capable of binding to the IGF-1R as well as its own IGF-2 receptor (IGF-2R). However, whilst the IGF-1R is capable of signal transduction, the IGF-2R is not and binding results in lysosomal degradation of IGF-2. This provides an indirect method of IGF-1 regulation by regulating the level of IGF-2 that is available to bind to the IGF-1R. Another set of related molecules that play a major role in the bioavailability of the IGFs are IGFBPs (Figure 2). These proteins bind to IGF-1 in circulation with an equal or greater affinity than that of the IGF-1R (k_d_ ~10^−10^ M vs. k_d_ ~10^−8^–10^−9^ M, respectively). There are six IGFBPs, binding approximately 98% of all circulating IGF-1 and serving as carrier proteins to regulate its transport and lengthen its otherwise comparatively short half-life [19,20] (Table 1) (Figure 2). Direct physical protein–protein interactions have been demonstrated for many of these proteins, summarized in Figure 3, which also shows other documented functional integrations between IGF-1 and its key interacting proteins. A convenient up-to-date summary of these is available from string-db.org. Of the six known IGFBPs, IGFBP-3 is by far the most common, accounting for around 80% of all bound IGF-1, typically forming a 150 kDa tri-molecular complex with it and the acid-labile subunit (ALS), known as the IGF-1-IGFBP-ALS complex [21]. By binding IGF-1, IGFBPs play a critical role in regulating its action and consequently cellular homeostasis. The up/down regulation of these important proteins is a complicated aspect of the IGF-signaling pathway and has yet to be comprehensively defined. Their action can be either IGF-dependent or independent and altered by various post-translational modifications. Such variations in IGFBP expression profiles have themselves been suggested as suitable markers for serious clinical conditions and even as potential therapeutics [22].

The superfamily of IGF binding proteins extends to a further ten proteins known as IGFBP-rPs that also bind IGFs, albeit at much lower affinities than the IGFBPs [23,24] (Table 1, Figure 2). The justification for their nomenclature has been questioned on the basis of their low affinity for IGFBPs, questionable ability to significantly influence IGF effects and the fact they share limited phylogenetic relationship with other members of the family [25]. IGFBP-rPs are involved in diverse biological functions, including growth regulation. IGFBP-rP1 and IGFBP-rP2 have been reported to increase during senescence of the prostate epithelium and in response to growth inhibitors (TGF-β1 and atRA) suggesting they are likely to negatively regulate growth, however the role of IGFBP-rP3 as a growth stimulator and/or protooncogene has been reported after preferential expression in cancerous cells [26]. rhIGFBP-rP3 has been shown to bind IGF-1, IGF-2 and insulin with low affinity and while synthesized by several malignant cell lines its ubiquitous presence in human biological fluids suggests that it could be involved in the regulation of cell growth in non-malignant tissues too [27,28]. 

Whilst the biological effect of IGF-1 is mediated by IGFRs, it is further affected by competitive action of other growth hormones. IGF-1 is implicated in a number of pathological conditions and is also a performance-enhancing drug (PED) widely abused in sports. The large number of IGFBPs, IGFBP-rPs as well as other carrier proteins affect bioavailability of IGF-1 in circulation and reduces significantly the levels of immunoreactive IGF-1 in serum samples, requiring multiple pre-treatment steps that reduce reproducibility and complicates interpretation of IGF-1 assay results. Having reviewed the IGF network of growth factors, IGFBPs, IGFBP-rPs and E peptides this review will summarize the role of IGF-1 as a marker in medical conditions and disease and will discuss issues related to the detection and quantification of bioavailable IGF-1.

## 2. IGF-1 as a Marker in Medical Conditions and Disease

Much of what is known about the function of IGF-1 has been established through studies of IGF-1 deficiencies [36,37] that can arise as a result of many different abnormalities in the hormones and receptors responsible for normal healthy growth [38,39,40]. Congenital IGF-1 deficiency may result from GH gene deletion, GH releasing hormone (GHRH) receptor defects and IGF-1 resistance. Primary IGF deficiency (PIGFD) is assigned to children exhibiting chronically low levels of IGF-1, despite normal levels of GH and no other underlying causes of poor growth [39]. Severe PIGFD (SPIGFD) is characterized by distinctive growth failure, with sufferers exhibiting IGF-1 levels below minus 3 standard deviations (SDs). The most common form of SPIGFD is known as Laron syndrome and results from a mutation to the *GHR* gene, rendering the affected individual insensitive to GH. Children diagnosed with Laron syndrome can be treated with recombinant IGF-1 [38,40]. Additional conditions under the umbrella of congenital IGF-1 deficiencies include a documented case of a 15-year-old boy suffering from severe prenatal and postnatal growth failure, sensorineural deafness and mental retardation due to a homozygous partial deletion of the IGF-I gene [41] as well as *IGF1R* genes defects [42]. Impairment of the GH-IGF-1 axis may also result from mutations to wider members of the IGF family including the *IGFALS* gene, as well as STAT5 defects that affect post-GHR signaling [43,44].

Acromegaly is a condition resulting from excess GH, typically caused by a benign tumor on the pituitary gland. Symptoms include abnormal enlargement of the hands and feet and in some cases the forehead, nose and jaw bones. In children the condition can lead to gigantism. Once diagnosed, treatment can be provided by removal of the pituitary tumor and/or medication to reduce the production of GH. Because IGF-1 is the prime mediator of GH in the body it has been used as a marker for the condition with up-regulation observed in patients of all ages who suffer from it [45]. The use of IGF-1 as a marker becomes even more important if the GH receptor antagonist pegvisomant is used as a treatment as this form of therapy means that circulating levels of GH are no longer suitable to monitor the status of the disease [46,47]. 

The pivotal role that IGF-1 plays in cell growth and proliferation, combined with the apparent protection against post-natal development of malignancies conferred by congenital IGF-1 deficiency, suggests a possible involvement of IGF-1 in the development of cancer [48]. Activation of IGF-1R following binding of IGF-1 has been shown to be involved in tumor cell growth and survival [49,50]. The positive correlation between IGF-1 expression and cancer has been widely reviewed with one study that included 18 years of follow-up reporting a 1.82-fold risk of cancer mortality among men who had a baseline IGF-1 level above 100 ng/mL compared with men with lower levels, a risk that was increased to 2.61 for men who had a baseline IGF-1 level over 200 ng/mL [51]. As a result of such studies, the IGF-1 signaling pathway became a target for new cancer therapeutics [52,53]. These approaches included anti-IGF-1R monoclonal antibodies (mAbs), IGF-1R tyrosine kinase inhibitors (TKIs), IGF-1/2 blocking mAbs, IGF ligand-TRAPs, recombinant IGFBPs and pregnancy-associated plasma protein-A (PAPP-A) inhibition [54,55]. Despite the sound pre-clinical reasoning, most efforts yielded disappointing results and were subsequently terminated [55,56]. More recently there has been cautious optimism among groups who believe that some studies were perhaps stopped prematurely and that while monotherapies might not be the way forward, combination therapies may hold greater promise [54,57,58,59].

Multiple studies have reported a positive association between IGF-1 and breast cancer [60,61,62,63]. More recently, complementary studies examined the associations between levels of IGF-1 in the blood and the chances of the disease developing in 206,263 women enrolled in the UK Biobank [64]. Women with IGF-1 concentrations in the top 20% were shown to have a 1.24-fold increased chance of developing breast cancer compared to those in the bottom 20%, after adjustments for factors including age, body mass index and concentrations of other hormones and proteins in the blood. Mendelian randomization was used to analyze data from 265 gene variants known to be associated with IGF-1 concentrations in 122,977 women with breast cancer and 105,974 women without cancer. Breast cancer risk increased by 1.05 for every additional genetically predicted 5 nmol/L of IGF-1. When comparing estrogen receptor positive (ER+) and negative (ER-) breast cancers separately IGF-1 was found to be only associated with an increased risk of ER+ breast cancer.

Prostate cancer is one of the most prevalent cancers in men, with most cases occurring in men over 50 years old. The suggestion that elevated IGF-1 levels may serve as a marker of risk has been long established [65,66], however several studies reported conflicting results, suggesting that IGF-1 was only a tumor marker rather than an etiologic factor in prostate cancer and as such was unsuitable as a long-term predictor of the disease [67,68,69,70]. More recently, the largest systematic review of studies on the association of IGF-1 with the risk of prostate cancer concluded that there is a 21% increased risk of prostate cancer per SD increase in IGF-1, with a stronger association with more aggressive and advanced cases [71]. After monitoring 200,452 men participating in the UK Biobank, Travia et al. [72] found that men with higher concentrations of IGF-1 are approximately 25% more likely to be diagnosed with prostate cancer compared to those with the lowest.

IGF-1 levels have been reported to be down-regulated in type 1 diabetes mellitus (T1DM) [73], yet up-regulated in type 2 diabetes mellitus (T2DM). The decrease in IGF-1 levels in T1DM happens despite the otherwise normal or elevated levels of GH, suggesting that GH-resistance is a factor. Elevated levels of serum Interleukin-8 (IL-8) in T1DM individuals with poor glucose control may contribute to an inflammatory response that has shown to lower IGF-1 levels in chronic inflammatory diseases [74]. While T2DM can be treated through improved diet and exercise as well as medications including insulin injections, the condition still results in a number of related complications. The most common complication among T2DM sufferers is an increased risk of cardiovascular mortality caused by atherosclerosis and equivalent to over 10 years of ageing. Obesity independent T2DM also increases risk of acute myocardial infarction and three quarters of all diabetic deaths are a result of coronary artery disease [75]. The elevated IGF-1 levels as a result of insulin-resistance are thought to be responsible for vascular deterioration and modulation of its levels may help to mitigate these adverse effects [76]. 

Non-alcoholic fatty liver disease (NAFLD) is a condition characterized by fat deposits in the liver that are not the result of excessive alcohol consumption. Like T2DM it is closely linked to insulin resistance and obesity and can lead to many of the same cardiovascular complications including advanced carotid atherosclerosis [77] and endothelial dysfunction. IGF-1 is known to play a protective role in ischemic heart and cardiovascular disease and atherosclerosis [78,79,80,81] and a study by Ichikawa et al. [82] confirmed down regulation in IGF-1 in NAFLD patients due to an inhibition of IGF-1 secretion via interleukin-1β (IL-1β), interleukin-6 (IL-6) and TNF-α. 

Sepsis is the systemic response to infection in which apoptosis of Küpffer cells is triggered by TNF-α that results in organ dysfunction. Through its activation of the phosphoinositol-3 kinase pathway and phosphorylation of the X-linked inhibitor of apoptosis protein (XIAP), IGF-1 acts to protect Küpffer cells and counter the potentially lethal effects of sepsis. IGF-1 levels have been shown to be decreased in children with Meningococcal sepsis and more so in those for whom the infection was fatal [83]. The potential for IGF-1 replacement as a treatment for both the prevention and management of sepsis has been demonstrated in mice where it improved overall survival rates through enhanced hepatic bacterial clearance [84] and in rats as a cognitive therapeutic when administered within 6 h of septic encephalopathy [85].

## 3. IGF-1 as a Performance-Enhancing Drug (PED): Doping in Sports

IGF-1 has gained a reputation as a popular doping agent in sport, implicated in many of the most high-profile doping cases in recent years including the Bay Area Laboratory Co-operative (BALCO) [86], Major League Baseball (MLB) [87] and Tour de France [88] scandals to name a few. The peptide’s stimulatory effect on muscle growth and tissue repair attracted the attention of athletes and trainers to whom increased strength and shorter recovery times are advantageous. Synthetic IGF-1, primarily intended for therapeutic treatment of conditions such as growth failure in children with SPIGFD [89], has been available since the late 1980s. Mecasermin is the generic name for recombinant human IGF-1 (rhIGF-1) derived from either yeast or E. coli and has been available since 1986 when it was developed by the Japanese company Fujisawa and later launched in 1995 marketed under the name Somazon. However, it was not for yet another decade that it received approval by the FDA for the US market when two companies, Tercica and Insmed, released their respective products Increlex and Iplex in 2005. Increlex was in fact Somazon, for which Tercica had acquired worldwide licensing rights outside Japan. Insmed’s Iplex however was an IGF-1/IGFBP-3 complex called mecasermin rinfabate (iPlex^TM^) [90] designed to better mimic IGF-1’s natural state and thus reduce side effects whilst also increasing its circulatory half-life [91,92]. Despite this, Insmed later agreed to withdraw its product from the US market in 2007 after it was found to infringe patents licensed by Tercica. 2007 also saw the European Medicines Agency (EMA) approve mecasermin for use in Europe for the exclusive treatment of children and adolescents who suffer from SPIGFD. Modified forms of the IGF-1 protein have been shown to have reduced affinity for the IGFBPs and as a result, increased potency [93]. IGF-1 DES is a recombinant and truncated form of IGF-1 produced in E.coli and lacking the first three amino acids of the N-terminus of the full peptide and thus also known as IGF-1 Des(1–3) and IGF-1 (4–70). This small modification results in a ~10-fold increase in potency in vivo [94]. Long arginine 3-IGF-1 (IGF-1 LR3) is another recombinant analogue of IGF-1 that has an arginine substitution at amino acid three and an additional thirteen amino acids at its N-terminus. This modification results in a significantly longer half-life, much lower affinity for the IGFBPs and, consequently, around a 3-fold increase in potency [95]. 

Shortly after IGF-1-containing products became more readily available, average 100 m and 200 m sprint times of elite athletes began to significantly decrease [96]. Whilst the authors acknowledged that other factors such as changes to training in certain populations may have contributed to the improvement, they also draw parallels with the way 5000 m running times began to dramatically fall a few years after erythropoietin (EPO) was introduced to the market. Similarly, a trend of improving female shot putt distances which started following the beginning of anabolic steroid use in female athletics, slowed with the advent of in-competition testing and then declined following the subsequent introduction of out-of-competition testing [96]. The introduction of FDA-approved pharmaceutical grade IGF-1 products in the form of Increlex and Iplex is likely to have seen an increase in rhIGF-1 abuse by athletes [97] and in the years that followed a number of newer IGF-1 compounds, analogues and products also became available, some even to the general public [98,99]. Such abuse as a performance-enhancing drug (PED) has led to exogenous IGF-1 being listed as a Prohibited Substance and officially outlawed by World Anti-Doping Agency (WADA), yet there is still no internationally recognized test for detecting rhIGF-1 abuse in sport. The increase in availability of rhIGF-1 came at a time when tests for GH also began to become more sophisticated, a development that is likely to have only furthered the adoption of IGF-1 as a doping agent for which most of the same desired effects from administering exogenous GH could be achieved. WADA currently employs two types of assay when testing for GH abuse: the so called Differential Isoforms Immunoassay that determines the ratio between the 22 kDa rhGH isoform and endogenous isoforms; and the hGH Biomarkers Test that is based on the measurement of IGF-1 and the N-terminal pro-peptide of type 3 collagen (P-III-P) as two markers shown to increase in a dose-dependent manner following GH administration [100].

The biomarker approach was developed by the GH-2000 group, a multi-center team led by Peter Sonksen at the appointment of the International Olympic Committee (IOC) [101]. The initial IGF-1 measurements were conducted using a commercial radioimmunoassay (RIA) kit produced by Nichols Institute Diagnostics. However, the kit was discontinued in 2005 and while it was advised that WADA pursue the development of an in-house assay to prevent such occurrences, the subsequent GH-2004 team chose to implement alternative commercial assays such as the DSL-5600 and Immunotech A15729 immunoradiometric assays, apply conversion factors to align results and make use of the reference range data that had been built using the prior assay [102]. The biomarker approach involving IGF-1 and P-III-P is capable of positive identification of doping up to 2 weeks post administration of GH (vs. just 12–24 h post-final dose with the isoforms method). Gender specific reference ranges of the two markers immediately following competition were established from studies carried out on over 800 elite athletes. The test was capable of detecting 100% of men using GH as a doping agent, with a false-positive rate better than 1:10,000 [103]. Whilst still successful, detection was less sensitive in females as they tend to exhibit greater resistance to the effects of GH. Further validation for factors such as sex, ethnicity and the effect of injury [103,104,105] increased sensitivity and saw the test introduced for the London 2012 Olympics leading to the disqualification of two Russian power lifters. 

Later the same group examined the applicability of these two markers for detection of exogenous rhIGF-1/IGFBP-3 in a randomized, double-blind, placebo-controlled study involving fifty-six recreational athletes [106]. While a relatively small rise in maximum marker levels was detectable (4-fold increase in males and females for IGF-1; 40–50% increase in women, 35–50% increase in men for P-III-P), the GH-2000 formulae applied for GH testing resulted in just 61% of women and 80% of men being correctly identified as having taken rhIGF-1. The study concluded that a greater sensitivity could be achieved (94% in both men and women) using a test based solely on IGF-1 concentrations.

## 4. Challenges of Monitoring IGF-1 Levels: Complications and Shortfalls of Existing Assays

The crucial role IGF-1 plays in human growth and development, the number of different clinical conditions that it is associated with, combined with the potential for its abuse in sports and its status as a marker for GH abuse, makes detecting its differential expression extremely important. At first glance, IGF-1 does indeed exhibit a number of characteristics desirable in a biomarker. It is generally considered to be stable for several days after collection [107,108] and during long-term storage at −25 °C for up to 12 months [109]. IGF-1 is insensitive to repetitive freeze-thaw cycles [110,111] and displays only minor circadian fluctuations, negating the need for repetitive standardized sampling procedures as is required with GH due to the pulsatile nature of its release and diurnal variation. A number of commercially available IGF-1 assay kits have long been available [112]. Despite their availability, interpreting IGF-1 results is still far from straightforward. The first major hurdle that became apparent shortly after the first IGF-1 assay was the discovery of IGFBPs, the presence of which significantly reduced the levels of immunoreactive IGF-1 in samples leading to misleadingly low results. The identification of IGFBPs was a major step forward in IGF-1 immunoassays as separating them prior to assay helped eliminate a significant source of error. Eliminating interference from IGFBPs can be achieved by removing them through effective yet laborious size exclusion chromatography at low pH but is more commonly achieved by acid-ethanol extraction and centrifugation. The latter involves dissociation of IGFBPs from IGF-1 by low pH, followed by ethanol precipitation and removal via centrifugation. However, this method is not perfect as centrifugation can result in either the undesired loss of IGF-1 from the sample or retention of residual IGFBPs in the supernatant [113]. As such, the technique is often combined with or centrifugation forgone in favor of, the addition of an “IGFBP blocking agent.” IGF-2 has proved useful in this regard, being added in excess to samples to reduce interference from residual IGFBPs but methods are not consistent across different IGF-1 assays [112]. The fact that established assays use different antibodies with differing epitope specificity has further contributed to the issue of contrasting results between assays [112,114]. Furthermore, it was not until 2008 that the World Health Organization (WHO) introduced the first International Standard for IGF-1 (02/254) [115], replacing the former International Reference Reagent (IRR 87/518) that had been widely used to calibrate the majority of IGF-1 assays up until this point despite the reagent’s protein content having been shown to be incorrectly assigned nearly a decade earlier [116]. The implications of this are that total IGF-1 concentrations reported by those assays were approximately 2-fold higher than actual values, calling into question the accuracy of a vast amount of IGF-1 data over a considerable time span. 

The interpretation of IGF-1 assay results is yet further complicated by the lack of a comprehensive set of reference ranges that define normal circulating levels across multiple populations. The Nichols Advantage immuno-chemiluminescent assay is widely referred to in the literature as having been the “gold standard” of IGF-1 testing and benefitted from a relative wealth of reference data across populations. However, its removal from the market when Nichols ceased trading in 2005 highlighted the importance of such reference ranges as researchers grappled with replacement kits that lacked the same level of normative data. While so-called conversion factors (CFs) have been reported, their reliability for comparing data between kits is questionable as demonstrated by Krebs et al. [117] who compared identical blood samples from 173 patients with GH deficiency using five different assays, as well as by Brabant & Wallaschofski [118] who deemed their use as infeasible. Furthermore, Khosravi et al. [119] reported that IGF-1 may well be more susceptible to post-sampling proteolysis than previously thought and that this could be one source of variability observed between results from multi-assay comparison tests that are otherwise highly correlated for fresh or well-preserved samples. As crucial as such reference value data sets are, their creation is no trivial task. While IGF-1 levels may exhibit minimal diurnal fluctuation, they are influenced by a number of other biological factors including age and pubertal stage, BMI extremes, pregnancy and to a lesser but still significant degree, sex and ethnicity. Following an expert workshop between members of The Growth Hormone Research Society, the International Federation of Clinical Chemistry and Laboratory Medicine, the International Society for IGF Research and the Pituitary Society, Clemmons [120] concluded that such a set of normative data should be based on the central 95% interval of a randomly selected set of individuals across all age groups from the background population, excluding those whose medical conditions or medication, are likely to affect their IGF-1 levels (e.g., those suffering from cirrhosis, diabetes or renal failure). The group also concluded that reference values should be presented at narrower intervals and in Tanner stages, for children and adolescents to reflect the greater change in IGF-1 levels at these ages, as well as sex-specific values between 6 and 18 years of age where gender-related variation is also greatest. 

## 5. Conclusions

IGF-1 and the related growth factors IGF-2 and insulin, their receptors and multiple IGF binding proteins constitute an extensive regulatory network involved in cell proliferation, differentiation and apoptosis. IGF-1 regulates normal growth during childhood and may exert a strong anabolic effect in adults. In its extremes, absence or excess of IGF-1 results in serious growth abnormalities, especially in children. IGF-1 is down-regulated in T1DM but is up-regulated in T2DM. IGF-1 elevation in adulthood may correlate with increased cancer risk, conversely, IGF-1 treatment can provide a therapeutic protection in sepsis. IGF-1 levels are indicative of multiple clinical conditions and many IGF-1 immunoaffinity-based assays are commercially available. How-ever, the bioavailability of IGF-1 depends heavily on multiple IGFBPs, IGFBP-rPs and an interplay between IGF-1, IGF-2, Insulin and their receptors, which complicates the interpretation of IGF-1 test results. 

The last few decades have brought major advances in the field of IGF-1 testing. Yet, the heterogeneity of existing assay characteristics and lack of comprehensive reference values, combined with previously inadequate calibration standards, together with cumbersome correction factors required to compare values between studies obtained with different assay kits, continue to affect the current unsatisfactory situation with IGF-1 testing. Consequently, the key unmet needs in this field relate to better IGF-1 detection and treatment options as well as a more precise understanding of IGF-1 involvement in chronic conditions combined with greater appreciation of how it can be administered acutely.

Recombinant IGF-1 and synthetic analogues were developed for therapeutic applications and started to appear on the market in the late 1980s. The stimulatory effect of IGF-1 administration on muscle growth and tissue repair was noted by a broad range of athletes leading to an increase in its abuse by them. This trend has slowed following the advent of testing and the prohibition of exogenous IGF-1 by WADA, yet there is still no internationally recognized test for detecting IGF-1 misuse in sport. While the introduction of the International Standard for IGF-1 (02/254) in 2009 was a step in the right direction, there clearly still exists quite some way to go if testing for IGF-1 is to produce results that can be interpreted and used with confidence. The standardization of sampling procedures and affinity reagents, as well as novel ways to eradicate the interference from IGFBPs, will enable significant progress towards achieving progress. Newly developed tests for IGF-1 will aim to identify those for whom IGF-1 levels are naturally high in addition to those who indulge in supplementation. Further advances will provide greater rigor in assays for a molecule that is intrinsically linked to so many important clinical conditions.

## Figures and Tables

**Table 1:** Human insulin-like growth factor-1 (IGF-1) and related other hormones and their binding proteins.

Hormones and Pro-Hormones	IGF Receptors	IGF Binding Proteins (IGFBPs)	IGFBP Related Proteins (IGFBP-rPs) ^1^
IGF-1 (P05019,	IGF-1R (P08069) ^2^	IGFBP-1 (P08833)	IGFBP-rP1/GFBP-7 (Q16270)
and Ea, Eb, Ec	IGF-2R (P11717) ^2^	IGFBP-2 (P18065)	IGFBP-rP2/IGFBP-8 (P29279)
pos. 119–195) ^3^	I-R (P06213) ^2^	IGFBP-3 (P17936)	IGFBP-rP3/IGFBP-9 (P48745)
IGF-2 (P01344)	^________________________^	IGFBP-4 (P22692)	IGFBP-rP4/IGFBP-10 (O00622)
Insulin (P01308)	IGFBPR [29]	IGFBP-5 (P24593)	IGFBP-rP5/L56/HtrA (Q92743)
^_______________________^		IGFBP-6 (P24592)	IGFBP-rP6/ESM-1 (Q9NQ30)
IGF-1 LR3 ^4^		^_______________________________^	IGFBP-rP7/WISP-2 (O76076)
des(1–3)IGF-1 ^5^		IGFBPL1 (Q8WX77) ^8^	IGFBP-rP8/WISP-1 (O95388)
Mecasermin ^6^		^_______________________________^	IGFBP-rP9/WISP-3 (O95389)
Mecasermin rinfabate ^7^		ALS (P35858) ^9^	IGFBP-rP10/KAZALD1 (Q96I82)

^1^ Nomenclature is based on [23,24]. ^2^ Two alternatively spliced forms of the insulin receptor (IR) may form two homodimeric I-Ra and I-Rb receptors or a number of heterodimer receptors combining the I-R and IGF-1R receptor subunits (IGF-1R/I-R). ^3^ IGF-1 pro-peptides Ea, Eb and Ec are generated from the same mRNA by alternative splicing. ^4^ Long-[Arg(3)]insulin-like growth factor-1. ^5^ 67 amino acids long, truncated IGF-1. ^6^ Recombinant human IGF-1 (rhIGF-1). ^7^ IGF-1/IGFBP-3 complex. ^8^ Insulin-like growth factor-binding protein-like 1 [30]. ^9^ Insulin-like growth factor-binding protein complex acid labile subunit.
